# Supplementary material for: HIVprotI: an integrated web based platform for prediction and design of HIV proteins inhibitors
Source: J Cheminform. 2018 Mar 9;10:12. doi: 10.1186/s13321-018-0266-y (PMC5845081; doi:10.1186/s13321-018-0266-y)
Supplement: Supplementary file 2 — Additional file 2. Source code of HIVProtI web server. [file 13321_2018_266_MOESM2_ESM.zip › HIVprotI_Source-code/hivprotI/weka-3-6-6/doc/weka/core/neighboursearch/covertrees/Stack.html]

Stack


---


|  |  |  |
| --- | --- | --- |
| **Overview** \| **Package** \| **Class** \| **Tree** \| **Deprecated** \| **Index** \| **Help** \| **Weka's home** | |  |
| PREV CLASS   NEXT CLASS | **FRAMES**    **NO FRAMES**     **All Classes** |  |
| SUMMARY: NESTED \| FIELD \| CONSTR \| METHOD | DETAIL: FIELD \| CONSTR \| METHOD |  |


---


## weka.core.neighboursearch.covertrees Class Stack<T>

```
java.lang.Object
  weka.core.neighboursearch.covertrees.Stack<T>
```

**Type Parameters:**: `T` - The type of elements to be stored in the stack.

**All Implemented Interfaces:**: java.io.Serializable, RevisionHandler

---

``` public class Stack<T> extends java.lang.Object implements java.io.Serializable, RevisionHandler ```

Class implementing a stack.

**Version:**
:   $Revision: 1.3 $

**Author:**
:   Alina Beygelzimer (original C++ code), Sham Kakade (original C++ code), John Langford (original C++ code), Ashraf M. Kibriya (amk14[at-the-rate]cs[dot]waikato[dot]ac[dot]nz) (Java port)

**See Also:**: Serialized Form

---

| **Field Summary** | |
| --- | --- |
| `java.util.ArrayList<T>` | `elements`             The elements inside the stack. |
| `int` | `length`             The number of elements in the stack. |


**Constructor Summary**

`Stack()`
  
          Constructor.

`Stack(int capacity)`
  
          Constructor.


| **Method Summary** | |
| --- | --- |
| `void` | `addAll(java.util.Collection c)`             Adds all the given elements in the stack. |
| `void` | `clear()`             Removes all the elements from the stack. |
| `T` | `element(int i)`             Returns the ith element in the stack. |
| `java.lang.String` | `getRevision()`             Returns the revision string. |
| `T` | `last()`             Returns the last element in the stack. |
| `T` | `pop()`             Pops (removes) the first (last added) element in the stack. |
| `void` | `push(Stack<T> v, T new_ele)`             Pushes the given element onto the given stack. |
| `void` | `push(T new_ele)`             Pushes the given element to the stack. |
| `void` | `replaceAllBy(Stack<T> s)`             Replace all elements in the stack with the elements of another given stack. |
| `void` | `set(int i, T e)`             Sets the ith element in the stack. |
| `java.util.List` | `subList(int beginIdx, int uptoLength)`             Returns a sublist of the elements in the stack. |

**Methods inherited from class java.lang.Object**

`equals, getClass, hashCode, notify, notifyAll, toString, wait, wait, wait`

**Field Detail**

### length

```
public int length
```

:   The number of elements in the stack.

---


### elements

```
public java.util.ArrayList<T> elements
```

:   The elements inside the stack.


**Constructor Detail**

### Stack

```
public Stack()
```

:   Constructor.

---


### Stack

```
public Stack(int capacity)
```

:   Constructor.

    **Parameters:**: `capacity` - The initial capacity of the stack.


**Method Detail**

### last

```
public T last()
```

:   Returns the last element in the stack.

    :   **Returns:**: The last element.

---


### element

```
public T element(int i)
```

:   Returns the ith element in the stack.

    :   **Parameters:**: `i` - The index of the element to return. **Returns:**: The ith element.

---


### set

```
public void set(int i,
                T e)
```

:   Sets the ith element in the stack.

    :   **Parameters:**: `i` - The index at which the element is to be inserted.: `e` - The element to insert.

---


### subList

```
public java.util.List subList(int beginIdx,
                              int uptoLength)
```

:   Returns a sublist of the elements in the
    stack.

    :   **Parameters:**: `beginIdx` - The start index of the sublist.: `uptoLength` - The length of the sublist. **Returns:**: The sublist starting from beginIdx and of length uptoLength.

---


### clear

```
public void clear()
```

:   Removes all the elements from the stack.

---


### addAll

```
public void addAll(java.util.Collection c)
```

:   Adds all the given elements in the stack.

    :   **Parameters:**: `c` - The collection of elements to add in the stack.

---


### replaceAllBy

```
public void replaceAllBy(Stack<T> s)
```

:   Replace all elements in the stack with
    the elements of another given stack.
    It first removes all the elements
    currently in the stack, and then adds all
    the elements of the provided stack.

    :   **Parameters:**: `s` - The stack whose elements should be put in this stack.

---


### pop

```
public T pop()
```

:   Pops (removes) the first (last added)
    element in the stack.

    :   **Returns:**: The poped element.

---


### push

```
public void push(T new_ele)
```

:   Pushes the given element to the stack.

    :   **Parameters:**: `new_ele` - The element to be pushed to the stack.

---


### push

```
public void push(Stack<T> v,
                 T new_ele)
```

:   Pushes the given element onto the given
    stack.

    :   **Parameters:**: `v` - The stack onto push the element.: `new_ele` - The element to push.

---


### getRevision

```
public java.lang.String getRevision()
```

:   Returns the revision string.

    :   **Specified by:**: `getRevision` in interface `RevisionHandler`
    :   **Returns:**: the revision


---


|  |  |  |
| --- | --- | --- |
| **Overview** \| **Package** \| **Class** \| **Tree** \| **Deprecated** \| **Index** \| **Help** \| **Weka's home** | |  |
| PREV CLASS   NEXT CLASS | **FRAMES**    **NO FRAMES**     **All Classes** |  |
| SUMMARY: NESTED \| FIELD \| CONSTR \| METHOD | DETAIL: FIELD \| CONSTR \| METHOD |  |


---
